# Unidirectional Palsy of Torsional Saccades in Ataxia Associated with Anti-GAD Antibody

**DOI:** 10.1007/s12311-026-01984-6

**Published:** 2026-03-24

**Authors:** Hyesoo Kwon, Hyo-Jung Kim, Jeong-Yoon Choi, Ji-Soo Kim

**Affiliations:** 1 Department of Neurology, Keimyung University Dongsan Hospital, Daegu, South Korea; 2 Biomedical Research Institute, Seoul National University Bundang Hospital, Seongnam, South Korea; 3 Department of Neurology, Seoul National University College of Medicine, Seoul, South Korea; 4 Department of Neurology, Dizziness Center, Clinical Neuroscience Center, Seoul National University Bundang Hospital, Seongnam, South Korea

**Keywords:** Glutamic acid decarboxylase, Ataxia, Ocular motor abnormality, Torsional saccades, Dizziness

## Abstract

Antibodies against glutamic acid decarboxylase (GAD) are associated with various neurological syndromes including cerebellar ataxia and ocular motor abnormalities. We report a 67-year-old man with progressive dizziness and imbalance, who exhibited spontaneous downbeat and torsional nystagmus, normal vertical saccades, and a unilateral loss of torsional saccades during head tilting. Serum anti-GAD antibody was elevated. Tests for other autoantibodies and genes responsible for spinocerebellar ataxia were negative. Brain MRI showed cerebellar vermis atrophy The selective deficit of ipsiversive torsional saccades, with preserved vertical saccades, implicates involvement of the unilateral rostral interstitial nucleus of the medial longitudinal fasciculus (riMLF) that generates torsional and vertical saccades. This case highlights a novel ocular motor finding in anti-GAD antibody-associated ataxia and expands the spectrum of eye movement abnormalities related to midbrain and cerebellar dysfunction.

**Supplementary Information:**

The online version contains supplementary material available at 10.1007/s12311-026-01984-6.

## Introduction

Antibodies against glutamic acid decarboxylase (GAD) have been associated with several neurological disorders including stiff-person syndrome, cerebellar ataxia, and epilepsy [1]. GAD is the enzyme that catalyzes the conversion of glutamate to γ-amino-butyric acid (GABA) and is also widely distributed in the central nervous system (CNS) [2]. Even though the pathogenic role of anti-GAD antibody remains unclear, a possible mechanism involves a change in the balance between glutamate and GABA, which may cause glutamate excitotoxicity [3]. While the entire CNS is affected by autoimmunity toward GAD, the cerebellum is one of the most vulnerable targets, along with the hippocampus [2]. Anti-GAD antibody is known to cause various ocular motor findings that include alternating skew deviation, horizontal or downbeat nystagmus, periodic alternating nystagmus, positional nystagmus, saccadic intrusions or oscillations, hypermetric saccades, and cross-coupled responses (downward corrective saccades) during horizontal head impulse tests [4, 5]. Herein, we report unilateral palsy of torsional saccades, a hitherto undescribed ocular motor abnormality, in a patient with anti-GAD antibody-associated ataxia.

## Case Report

**Fig. 1:**
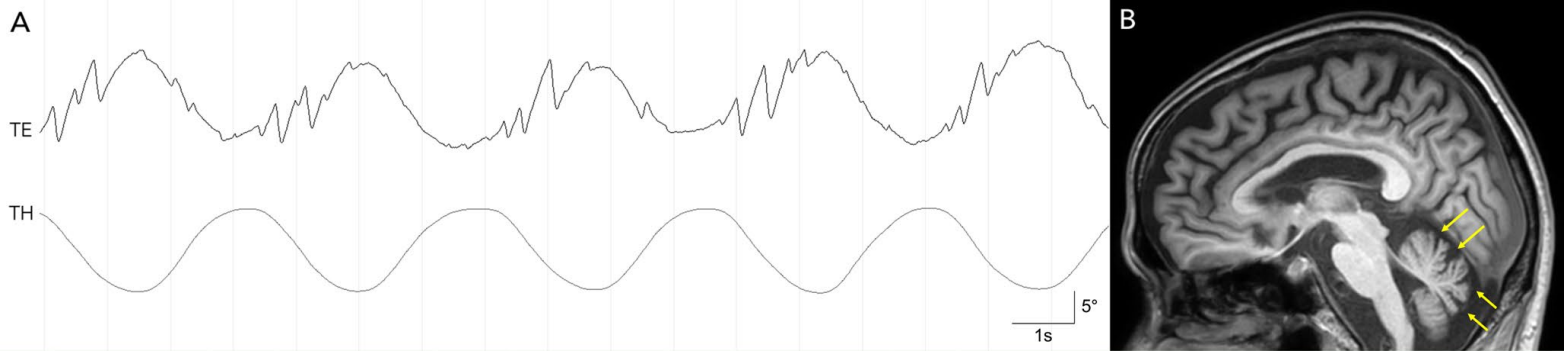
**A** Video-oculography shows a loss of clockwise torsional quickphases (saccades) during rightward head tilting. Upward deflection in each trace indicates a clockwise eye motion (the upper poles of the eyes moving toward the right ear) or rightward head tilting. TE: torsionalposition of the eye, TH: torsional position of the head. **B** T1-weightedsagittal MRI shows diffuse atrophy involving the cerebellar vermis (yellow arrows)

A 67-year-old man presented with progressive dizziness and imbalance for 3 months. He had a history of distal gastrectomy due to stomach cancer about 20 years before. On neurological examination, he showed mild appendicular dysmetria on both sides and instability during tandem gait. The eyes were orthotropic with normal eyelid and pupillary functions. Further ocular motor evaluation using video-oculography showed spontaneous downbeat and counterclockwise (upper poles of the eyes beating toward the left ear) torsional nystagmus in primary gaze (Video 1). The downbeat nystagmus increased during leftward and downward gazes. The torsional nystagmus increased during leftward gaze. Downward smooth pursuit was impaired. Horizontal and vertical saccades were normal. Head turning to either side while supine induced persistent geotropic nystagmus along with an augmentation of downbeat nystagmus. Downbeat nystagmus also increased during Dix-Hallpike maneuver in either direction. Video head impulse tests were normal for all six semicircular canals. During head oscillation in the roll plane, the clockwise (upper poles of the eyes beating toward the right ear) torsional quick phases were near completely abolished when the head was tilted rightward, thus giving rise to a distinct asymmetry between the sides (Fig. 1A, Video 2). The Scale for the Assessment and Rating of Ataxia (SARA) was measured at 2.5. Brain MRIs documented diffuse atrophy involving the cerebellar vermis without a structural lesion in the midbrain (Fig. 1B). No tumor was detected on chest or abdomen CT scans. The patient had no history of other autoimmune diseases. Serum thiamine was measured at 2.8 µg/dL (reference range, 2.0–7.2 µg/dL) while serum vitamin B12 was not measured. Paraneoplastic antibodies (anti-Hu, anti-Ri, anti-Yo, anti-amphiphysin, anti-CV2, anti-Ma2/Ta, anti-recoverin, anti-SOX1, and anti-titin) and genetic testing for spinocerebellar ataxia (SCA1, 2, 3, 6, 7, and 17) and dentatorubral-pallidoluysian atrophy were all negative. Genetic testing for SCA27B was not performed. However, serum anti-GAD antibody measured using a radioimmunometric assay specific for GAD65 was found to be elevated at 9.23 U/mL (reference range, 0–1.0 U/mL). Cerebrospinal fluid analysis was not performed. Immunotherapy was initiated with azathioprine 50 mg per day, but without improvement for 5 months of medication. Subsequent trials of intravenous cyclophosphamide and immunoglobulin also failed to reverse the clinical course. Serum anti-GAD antibody remained elevated at 12.73–17.00 U/mL during the follow-up of 3 years.

## Discussion

Our patient with ataxia and anti-GAD antibody showed a near complete loss of clockwise torsional saccades in the presence of normal vertical saccades. In monkeys, lesions involving unilateral rostral interstitial nucleus of the medial longitudinal fasciculus (riMLF) result in a loss of ipsiversive torsional rapid eye movements with a sparing of vertical saccades [6], like in our patient. The burst neurons for generation of torsional and vertical saccades are located in the riMLF [7]. A previous report described supranuclear paresis of vertical gaze, predominantly downward, in a patient with stiff-person syndrome associated with anti-GAD antibody [8]. To the best of our knowledge, however, unilateral loss of torsional saccades has not been described in humans. Instead, a patient with a lesion presumed to have involved unilateral riMLF showed an abnormal contraversive torsional deviation of the eyes during vertical saccades [9].

Our patient also showed counterclockwise torsional nystagmus along with downbeat nystagmus. In a previous study on 11 patients with a stroke involving the rostral midbrain, all patients initially showed spontaneous nystagmus with a torsional component, mostly contraversive, during primary gaze [10]. Indeed, a unilateral lesion of the riMLF causes contraversive torsional nystagmus while a unilateral lesion of the interstitial nucleus of Cajal gives rise to ipsiversive torsional nystagmus [7]. Along with a loss of ipsiversive torsional quick phases, the spontaneous contraversive torsional nystagmus observed in our patient indicates a lesion involving ipsilateral riMLF.

Our patient showed persistent geotropic nystagmus on head turning to either side while supine. Patients with anti-GAD antibodies may show various patterns of positional nystagmus that include paroxysmal geotropic, apogeotropic, and vertical (either downbeat or upbeat) nystagmus [5, 11, 12]. Along with spontaneous downbeat nystagmus, the presence of positional nystagmus indicates an involvement of the cerebellum that is the most vulnerable organ in anti-GAD antibody syndrome.

## Conclusion

In conclusion, anti-GAD antibody may cause a torsional saccadic palsy in addition to cerebellar dysfunction by involving the riMLF that is responsible for vertical and ipsiversive torsional saccades in the mesodiencephalic junction. Given the normal vertical saccades observed in our patient, a loss of ipsiversive torsional saccades may be a more sensitive sign than vertical saccadic slowing in lesions involving unilateral riMLF.

## Supplementary Information

Below is the link to the electronic supplementary material.

Video 1. Video-oculography shows spontaneous downbeat and counterclockwise (upper poles of the eyes beating toward the left ear) torsional nystagmus in primary gaze, which increases without visual fixation. H: horizontal position of the right eye, T: torsional position of the right eye, V: vertical position of the right eye .

Video 2. Head oscillation in the roll plane shows a loss of clockwise torsional quick phases (saccades) during rightward head tilting. TE: torsional position of the eye, TH: torsional position of the head.

## Data Availability

Anonymized data will be shared by request from any qualified investigator.
